# Preparation of self-assembly silica redox nanoparticles to improve drug encapsulation and suppress the adverse effect of doxorubicin

**DOI:** 10.5599/admet.1845

**Published:** 2023-07-04

**Authors:** Minh-Dat Quoc Tang, Nhu-Thuy Trinh, Dung Vu, Thu-Ha Thi Nguyen, Hung Thanh Dong, Toi Van Vo, Long Binh Vong

**Affiliations:** 1 School of Biomedical Engineering, International University, Ho Chi Minh 700000, Vietnam; 2 Vietnam National University Ho Chi Minh City (VNU-HCM), Ho Chi Minh 700000, Vietnam

**Keywords:** Chemotherapy, reactive oxygen species, ROS scavengers, micelles nanoparticles, nanomedicine

## Abstract

**Background and Purpose:**

The utilization of doxorubicin (DOX) in clinal trials is also challenging owing to its adverse effects, including low oral bioavailability, generation of reactive oxygen species (ROS), cardiotoxicity, and epithelial barrier damage. Recently, scavenging of ROS reduced the cytotoxicity of DOX, suggesting a new approach for using DOX as an anticancer treatment. Thus, in this study, non-silica and silica redox nanoparticles (denoted as RNP^N^ and siRNP, respectively) with ROS scavenging features have been designed to encapsulate DOX and reduce its cytotoxicity.

**Experimental Approach:**

DOX-loaded RNP^N^ (DOX@RNP^N^) and DOX-loaded siRNP (DOX@siRNP) were prepared by co-dissolving DOX with RNP^N^ and siRNP, respectively. The size and stability of nanoparticles were characterized by the dynamic light scattering system. Additionally, encapsulation efficiency, loading capacity, and release profile of DOX@RNP^N^ and DOX@siRNP were identified by measuring the absorbance of DOX. Finally, the cytotoxicity of DOX@RNP^N^ and DOX@siRNP against normal murine fibroblast cells (L929), human hepatocellular carcinoma cells (HepG2), and human breast cancer cells (MCF-7) were also investigated.

**Key results:**

The obtained result showed that RNP^N^ exhibited a pH-sensitive character while silanol moieties improved the stability of siRNP in physiological conditions. DOX@RNP^N^ and DOX@siRNP were formed at several tens of nanometers in diameter with narrow distribution. Moreover, DOX@siRNP stabilized under different pH buffers, especially gastric pH, and improved encapsulation of DOX owing to the addition of silanol groups. DOX@RNP^N^ and DOX@siRNP maintained anticancer activity of DOX against HepG2, and MCF-7 cells, while their cytotoxicity on L929 cells was significantly reduced compared to free DOX treatment.

**Conclusion:**

DOX@RNP^N^ and DOX@siRNP could effectively suppress the adverse effect of DOX, suggesting the potential to become promising nanomedicines for cancer treatments.

## Introduction

Doxorubicin (DOX, also known as Adriamycin) has been widely used to treat numerous cancer types since its approval by the FDA in 1974 [1]. The most well-known anticancer mechanism of DOX is the interaction with the topoisomerases II, leading to interference with the DNA replications. Additionally, DOX also induces reactive oxygen species (ROS) via nonenzymatic and enzymatic pathways [2,3]. Consequently, these free radicals cause intracellular oxidative stress, and damage cellular membranes and DNA, although this mechanism is not considered to play a major role in killing cancer cells [4]. Unfortunately, DOX is non-specific distribution due to its low molecular weight drug nature, resulting in myocyte, myelocyte, and hematocyte toxicities [5-7]. DOX-induced damage on intestinal epithelium has also been reported [8]. Besides, the low oral bioavailability of DOX was reported at 1 % due to its absorption through the paracellular pathway [9]. Lowering the cytotoxicity of DOX and enhancement of its oral bioavailability have gained attention to extend the clinical utility of DOX. Recently, scavengers of free radicals showed prevention of DOX-induced cardiotoxicity [10], implying the important role of ROS scavengers in reducing the adverse effects of DOX.

Nanoparticles (NPs) have been widely developed as carriers for drug delivery systems (DDS) to decrease unwanted diffusion, increase drug bioavailability, and control pharmacokinetics [11-13]. Oral administration is one of the most popular pathways to deliver NPs owing to its unique advantages like non-invasion, painlessness, easy administration, and patient compliance. The various NPs, such as liposomes, metal, and polymeric NPs, have been designed for oral drug delivery. However, most techniques still face numerous challenges, such as gastric juice influencing the aggregation state of liposomes [14], decreasing migration, retention in the intestinal mucus layer, and low blood uptake of the therapeutic agents. Moreover, the activation of various enzymes by oral administration also causes instability and leakage of payload, suggesting an efficient reduction of drug carriers and toxicity owing to the exposure of the drug on the gastrointestinal (GI) tract. Therefore, the requirements for oral DDS of cancer treatment are stable dispersion, drug encapsulation in the harsh GI tract, and low toxicity. Recently, polymeric NPs have been applied to improve the oral bioavailability of chemotherapy. For example, DOX-encapsulated enoxaparin sodium-PLGA hybrid NPs significantly increased retention time in the pharmacokinetics study [15]. Moreover, polymeric micelles, a type of polymeric NPs with a core-shell structure, can increase the stability, and permeability in the intestinal epithelium and reduce the degradation of anti-cancer drugs. For instance, DOX-loaded polymeric micelles significantly improved the intestinal absorption rate and systemic circulation time as compared to free DOX [16]. Thus, micelles NPs can be considered potential anti-cancer DDS in oral administration.

Recently, we have designed two types of core-shell antioxidant NPs, pH non-sensitive (RNP°) and pH-sensitive (RNP^N^), as ideal oral DDS to solve these problems. RNP° and RNP^N^ were prepared through self-assembly amphiphilic block copolymers containing ROS scavengers (nitroxide radicals, TEMPO) at the side chains of the hydrophobic segment. The ROS-scavenging ability of RNPs is an important factor in decreasing the DOX-induced oxidation stress. Both RNPs indicated highly dispersible and biocompatible properties with a long half-life in circulation compared to free nitroxide radicals. In addition, RNPs have been researched as possible treatments for solid tumours [17,18]. Although RNP° prolonged blood circulation as compared to RNP^N^ [19,20], RNP° was not appropriated in encapsulating drugs inside the core [21]. In contrast, gastric pH is also challenging for RNP^N^ due to its pH sensitivity caused by the protonation of amino groups on the hydrophobic core. Therefore, we have developed silica-installed redox NPs (siRNP) through sol-gel chemistry by the hydrolysis and condensation of tetraethyl orthosilicate (TEOS) with the presence of ammonia during the preparation of RNP^N^. Crosslinking of silanol groups is expected to improve the stability of NPs and the drug encapsulation efficacy via adsorption on the silica surface and electrostatic interactions between DOX and silica. In this work, we studied the impacts of RNP^N^ and siRNP loading DOX (denoted as DOX@RNP^N^ and DOX@siRNP, respectively) to reduce the side effects of DOX on normal cells and to maintain the cytotoxicity properties of DOX on cancer cells. The obtained result indicated that NPs were nano-size distribution and high dispersion in the physiological conditions. Moreover, the addition of silanol groups significantly improved the encapsulation of DOX and the stability of RNP^N^ in gastric pH. Compared to free DOX treatment, the DOX@RNP^N^ and DOX@siRNP showed lower cytotoxicity against murine fibroblast cells (L929), while they maintained the cytotoxicity on human breast cancer cells (MCF-7) and human hepatocellular carcinoma cells (HepG2).

## Experimental

### Chemicals

Dimethylformamide (DMF, Sigma-Aldrich, USA), permeable membrane tube (MWCO 3.5 kDa, Spectrum Laboratories Inc., Japan), tetraethyl orthosilicate (TEOS, Sigma-Aldrich, USA), ammonia (NH_3_, China), Doxorubicin hydrochloride (DOX, Wako, Japan), 3-(4,5-dimethylthiazol-2-yl)-2,5-diphenyltetrazolium bromide (MTT, Roche Diagnostics, Japan), dimethyl sulfoxide (DMSO, China), Dulbecco’s Modified Eagle’s Medium (DMEM, Gibco, USA), fetal bovine serum (FBS, Sigma-Aldrich, USA), and antibiotics (a mixture of penicillin, streptomycin, and neomycin, Sigma-Aldrich, USA) were purchased.

### Preparation and characterization of RNP^N^ and siRNP

**Figure 1.**
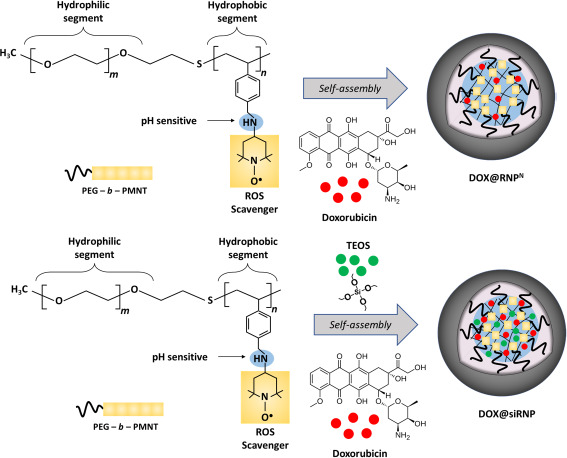
Illustration of DOX@RNP^N^ and DOX@siRNP preparation. DOX@RNP^N^ was prepared by co-dissolving amphiphilic copolymers PEG-*b*-PMNT and DOX while DOX@siRNP was similarly prepared, except TEOS was added to form silica NPs under the catalyst of NH_3_ during the stirring process.

RNP^N^ was prepared by self-assembly amphiphilic copolymers PEG*-b-*PMNT, as reported in previous research [18]. Briefly, 15 mg of PEG-*b*-PMNT were dissolved in 1 mL of DMF and then stirred on a magnetic stirrer. The mixture was then put into a semi-permeable membrane tube before being dialyzed against distilled water for 24 h. To prepare siRNP, a similar method was conducted, except that 50 μL of TEOS and 50 μL of NH_3_ were added during the stirring process (Figure 1). The average size, polydispersity index (PdI), and size distribution of RNP^N^ and siRNP were then characterized by the dynamic light scattering (DLS, Malvern Zetasizer, UK) system.

### The stability of RNP^N^ and siRNP

Phosphate-buffered saline (PBS, 10 mM) was prepared at different pHs, including 3.0, 6.5, and 7.4, to mimic the pH levels of the gastric, tumour extracellular, and physiological environments, respectively. RNP^N^ and siRNP were then diluted in the prepared buffers. The stability of RNP^N^ and siRNP was evaluated by measuring the change in light scattering intensity using the DLS system at 25 °C for 2 h.

### Preparation and characterization of DOX@RNP^N^ and DOX@siRNP

DOX@RNP^N^ and DOX@siRNP were prepared by co-dissolving 1 mg of DOX and 15 mg of PEG-*b*-PMNT in 1 mL of DMF. Similar steps were followed in the preparation of RNP^N^ and siRNP, respectively (Figure 1). Additionally, various ratios of TEOS:NH_3_ (200 μL TEOS: 200 μL NH_3_, 100 μL TEOS: 100 μL NH_3_, 50 μL TEOS: 50 μL NH_3_, 100 μL TEOS: 50 μL NH_3_, 200 μL TEOS: 50 μL NH_3_) were examined to optimize the size of DOX@siRNP. The DLS system was used to characterize the average size, PdI, and size distribution of DOX@RNP^N^ and DOX@siRNP.

### Drug encapsulated efficiency and loading capacity of DOX@RNP^N^ and DOX@siRNP

The encapsulation efficiency (*EE* / %) and loading capacity (*LC* / %) of DOX@RNP^N^ and DOX@siRNP were investigated based on the amount of loaded DOX evaluated by a microplate reader (Thermo Fisher Scientific, USA) with a calibration curve of DOX measured absorbance at 480 nm. The *EE* and *LC* were calculated by using equation (1) and (2):


(1)

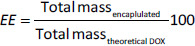




(2)

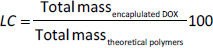



### Release profile of DOX@RNP^N^ and DOX@siRNP

2 mL of DOX@RNP^N^ or DOX@siRNP were loaded into separate dialysis bags and placed in different beakers under stirring conditions, each with distinct pH including 3, 6.5, and 7.4. At predetermined time points (0, 1, 3, 6, 9, 20, and 24 h), samples were collected and the absorbance at 480 nm was measured to evaluate the amount of drug released from the NPs.

### Cytotoxic assay

The murine fibroblast cells (L929), human breast cancer cells (MCF-7), and human hepatocellular carcinoma cells (HepG2) were obtained from the American Type Culture Collection (ATCC, USA). The cells were cultured in DMEM containing 10 % FBS and 1 % antibiotics (a mixture of penicillin, streptomycin, and neomycin) at 37 °C and 5 % CO_2_. The cells were then seeded on 96-well plates (10^4^ cells/well) and incubated for 24 h at 37 °C and 5 % CO_2_. The tested samples, including RNP^N^, siRNP, DOX, DOX@RNP^N^, and DOX@siRNP, were added to each well to obtain final concentrations of DOX at 0.25, 0.5, 1, 2.5, and 5 μg/mL, and incubated for 24 h. After that, the medium was removed, and MTT solution was added to each well and continuously incubated for 4 h. Finally, DMSO was added to dissolve crystals of formazan inside the cells, and the plates were measured absorbance at 540 nm by a microplate reader (Thermo Fisher Scientific, USA). The cell viability (%) was calculated by using equation (3):


(3)

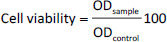



where OD_sample_ is the absorbance of the sample and OD_control_ is the absorbance of the solvent.

### Statistical analysis

Statistical analysis was performed using one-way analysis of variance (ANOVA) to determine significant differences among various groups. A *p*-value <0.05 was considered statistically significant. Data were presented as the mean ± standard deviation (SD).

## Results and discussion

### Characterization of RNP^N^ and siRNP

**Figure 2.**
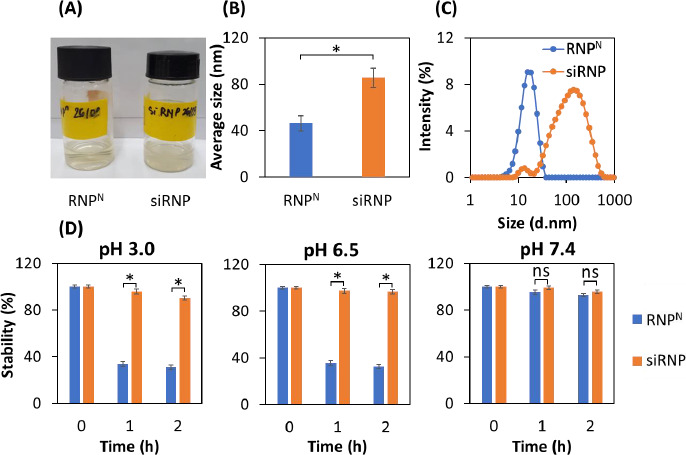
Characterization of RNP^N^ and siRNP. The micelles were obtained by self-assembly amphiphilic copolymers PEG-*b*-PMNT and the addition of silanol groups through the dialysis method for RNP^N^ and siRNP, respectively, and then characterized by the DLS system. (A) The photographs after dialysis for 24 h. (B) Average size. (C) Size distribution. (D) The stability of RNP^N^ and siRNP in PBS with different pHs, including 3, 6.5, and 7.4 simulated for the gastric, tumor extracellular, and physiological environments, respectively. Data were presented as the mean ± standard deviation (SD); *n* =3; *p* < 0.05 (*); ns = non-significant.

RNP^N^ and siRNP were prepared by self-assembly amphiphilic copolymers PEG-*b*-PMNT in DMF, followed by dialysis against distilled water for 24 h to remove the organic solvent. The results showed that both RNP^N^ and siRNP were transparent (Figure 2A), with average sizes of 45.5 and 85.5 nm, respectively (Figure 2B). Furthermore, the PdI of both NPs was less than 0.3, indicating a narrow size distribution. The increase in the size of siRNP as compared to RNP^N^ might be explained by a sol-gel reaction of TEOS in the RNP^N^ core [22]. The size of siRNP was approximately 85 nm after the condensation reaction, suggesting no significant aggregation (Figure 2C), even though silica particles formed by sol-gel can reach up to 2000 nm. We next examined the influence of different pH buffers on the stability of NPs [23]. As shown in Figure 2D, RNP^N^ disintegrated under acidic pH (3.0 and 6.5) and stabilized at pH 7.4. In contrast, siRNP was stable regardless of pH change. This might be explained that the amino groups of the PMNT segment disintegrated due to their protonation at low pH. However, adding silica would maintain the structure of NPs at acidic pH, resulting in enhanced stability.

### Characterization of DOX@RNP^N^ and DOX@siRNP

**Figure 3.**
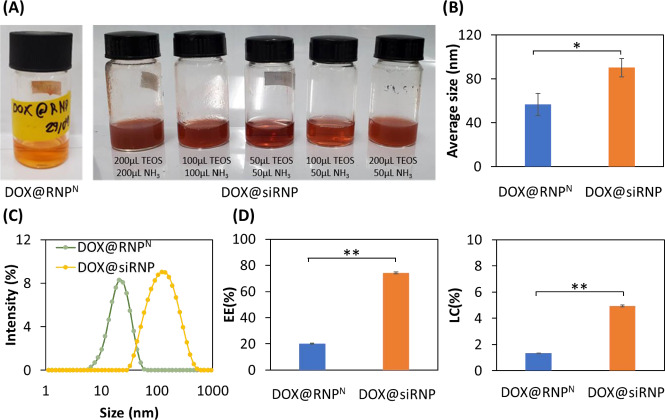
Characterization of DOX@RNP^N^ and DOX@siRNP. The method for preparation of DOX@RNP^N^ and DOX@siRNP was similar to the method of RNP^N^ and siRNP except for the incorporation of DOX during the stirring process. (A) The photographs of DOX@RNP^N^ and DOX@siRNP with different ratios of TEOS:NH_3_ after dialysis for 24 h. (B) Average size. (C) Size distribution. (D) The EE and LC of DOX@RNP^N^ and DOX@siRNP were obtained by measuring the absorbance at 480 nm. Data were presented as the mean ± standard deviation (SD); *n* =3; *p* < 0.05 (*); *p* < 0.01 (**).

DOX@RNP^N^ and DOX@siRNP were similarly prepared as RNP^N^ and siRNP, respectively, except for the addition of DOX during the preparation process. Different concentrations of NH_3_ might result in a change in the size of silica NPs despite its catalyzation role in sol-gel reactions [24]. Thus, different ratios of TEOS:NH_3_ were examined to prepare DOX@siRNP with the nano-size distribution. As shown in Figure 3A, DOX@RNP^N^, DOX@siRNP with ratios of 50 μL TEOS: 50 μL NH_3_ and 100 μL TEOS: 50 μL NH_3_ were transparent, resulting in the nano-size distribution as compared to other samples with turbidity in solution and micro-size distribution (data not shown). Hence, DOX@siRNP with a ratio of 50 μL TEOS: 50 μL NH_3_ was chosen for further experiments because of its suitable size. As compared to NPs, the size of both DOX@RNP^N^ and DOX@siRNP significantly increased to 56 and 90 nm, respectively, implying the encapsulation of DOX in NPs (Figure 3B). The critical cut-off point for endocytosis has been estimated from 20 to 500 nm for M cells and from approximately 50 to around 100 nm for enterocyte cells [25]. Therefore, DOX@RNP^N^ and DOX@siRNP showed the potential to increase the oral bioavailability of DOX. Moreover, the PdI of both NPs was around 0.3, indicating the narrow size distribution even after drug encapsulation (Figure 3C). Conventionally, polymeric NPs exhibit a low EE by physical entrapment of the drug (less than 10 %) [26-28]. For example, the LC of DOX-loaded silica NPs was reported at 4.8 % [29]. As shown in Figure 3D, the EE was 20.3 and 74.9 %, and the LC was 1.5 and 5 % for DOX@RNP^N^ and DOX@siRNP, respectively. This result indicates the appropriate design of the DOX encapsulation because the silanol groups in the core of siRNP significantly improved the EE and LC of DOX. This result could be explained by the pKa of the amino group in DOX being around 9.9, thus most DOX molecules are in the positively charged form at physiological pH [30]. Consequently, silanol moieties possessing a negatively charged surface could interact with DOX by electrostatics, suggesting the enhancement in EE and LC of DOX@siRNP.

### Release profile of DOX@RNP^N^ and DOX@siRNP

**Figure 4.**
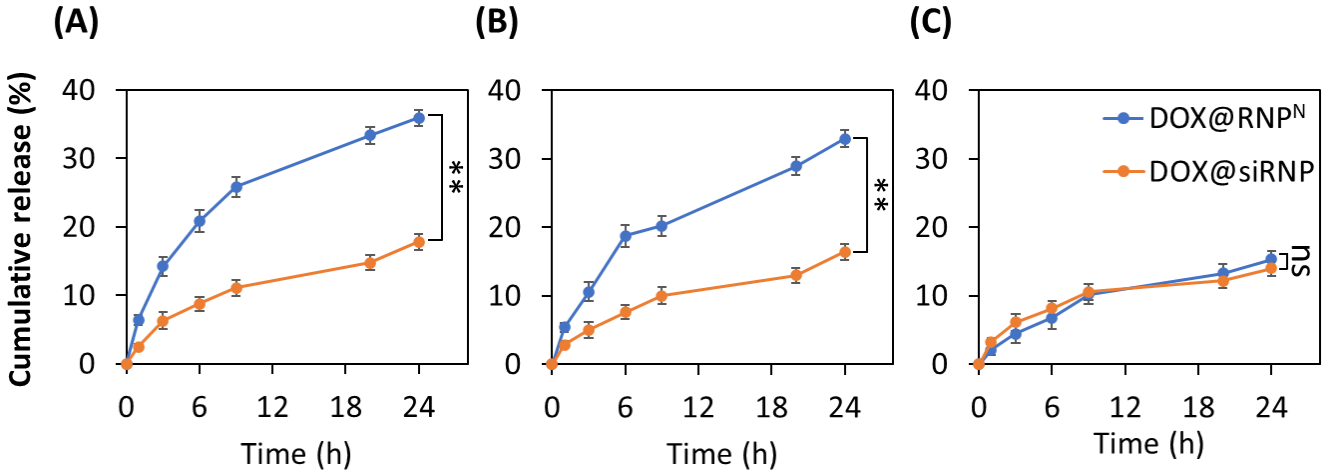
The drug release of DOX@RNP^N^ and DOX@siRNP. The release of DOX in PBS with various pH including 3.0, 6.5, and 7.4 was identified by measuring the absorbance at 480 nm. (A) pH 3.0. (B). pH 6.5. (C) pH 7.4. Data were presented as the mean ± standard deviation (SD); n =3; p < 0.01 (**); ns = non-significant.

The release kinetics of DOX@RNP^N^ and DOX@siRNP were evaluated under different pH buffers, including pH 3.0, 6.5, and 7.4, which mimic the gastric, tumour extracellular, and physiological pH, respectively. Gastric pH is one of the challenging barriers for oral drug delivery systems. As shown in Figure 4A, DOX@siRNP was released significantly slower than DOX@RNP^N^ (17.8 % compared to 35.9 %) after 24 h, even though both experienced protonations of amino groups at the hydrophobic core. However, more than 80 % of DOX@RNP^N^ and 90 % of DOX@siRNP were retained after 6 h, suggesting their potential for *in vivo* application as they can withstand the maximal transit time of the stomach in the human body. The extracellular pH of tumors, which commonly drops around 6.5-7.0, is mainly caused by anaerobic glycolysis in hypoxia [31]. As shown in Figure 4B, the release profiles of DOX@siRNP and DOX@RNP^N^ at pH 6.5 were 16.4 and 33.0 %, respectively. These results are consistent with the previous stability study. It is evident that DOX@siRNP was less impacted by acidic pH than DOX@RNP^N^ due to the ability of nano-silica to stabilize the structure. After 24 h at physiological pH, DOX@RNP^N^ and DOX@siRNP leaked at 14 and 15 %, respectively (Figure 4C). Similarly, several studies have reported that the release profile of micelle NPs with pH-sensitive groups is higher at acidic pH than at neutral pH [32,33].

### In vitro cytotoxicity

**Figure 5.**
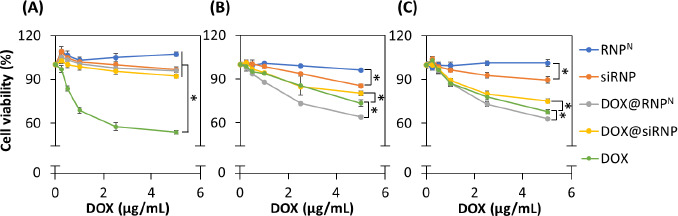
The cytotoxicity on various cells. The cell viability was measured by MTT assay. (A) Murine fibroblast cells (L929). (B) Human breast cancer cells (MCF-7). (C) Human hepatocellular carcinoma cells (HepG2). Data were presented as the mean ± standard deviation (SD); n =3; p < 0.05 (*).

The cytotoxicity of NPs was assessed through MTT assay on normal murine fibroblast cells (L929) and cancer cells, including human breast cancer cells (MCF-7) and human hepatocellular carcinoma cells (HepG2). In addition to the well-known anticancer mechanism, which involves interaction with the topoisomerases II to interfere with DNA replication, it also induces the over-expression of free radicals to kill cells. Therefore, RNP^N^ with TEMPOL, a ROS scavenger in the hydrophobic core, was used to encapsulate DOX to reduce its side effects in an *in vitro* model. As shown in Figure 5A, DOX indicated dose-dependence, with approximately 50 % of L929 cell death at a concentration of 5 μg/mL DOX, while the NPs significantly enhanced cell viability, almost equal to the control sample. Despite dose-dependent toxicity observed in DOX@RNP^N^ and DOX@siRNP, there was more than 90 % of cell viability after 24 h of incubation due to the scavenging ability of TEMPO groups. In addition, silica NPs have been reported to cause cytotoxicity due to the overproduction of ROS [34], leading to lower cell viability of siRNP compared to RNP^N^. However, siRNP treatment exhibited more than 90 % of cell viability, suggesting the biosafe profile of NPs. Similarly, anticancer effects of DOX were observed on human breast cancer cells (MCF-7) and human hepatocellular carcinoma cells (HepG2), with dose dependence (Figure 5B and Figure 5C). Remarkably, DOX@siRNP caused fewer cell deaths (around 20 and 25 %) compared to (27 and 32 %) at 5 μg/mL of free DOX treatment against MCF-7 and HepG2, respectively. This result may be due to DOX being kept inside the core of siRNP, resulting in less exposure to cells, although encapsulation of DOX in NPs was expected to be more effective at entering cells. In contrast, the anticancer effect caused by DOX@RNP^N^ (5 μg/mL of DOX) was 36 and 37 % for MCF-7 and HepG2, respectively. It is obvious that the cumulative release of DOX@RNP^N^ was faster as compared to DOX@siRNP, resulting in higher cytotoxicity when entering cells.

## Conclusions

In this study, non-silica and silica redox NPs (RNP^N^ and siRNP, respectively) with ROS scavenging moieties on hydrophobic side chains were prepared to encapsulate DOX to reduce its adverse effects. The DOX@RNP^N^ and DOX@siRNP were successfully prepared with several tens nm distributions and stable dispersion. The obtained results indicated that the addition of silanol groups significantly improved the stability of RNP^N^ in acidic pH and the encapsulation efficacy of DOX into siRNP. Due to its pH-sensitive character, DOX@RNP^N^ exhibited a faster release of DOX under acidic pH environments as compared to DOX@siRNP. Both DOX@RNP^N^ and DOX@siRNP suppressed the cytotoxicity of DOX against normal fibroblast cells while they exhibited the anticancer activity to induce cell death in liver cancer and breast cancer cell lines. Therefore, DOX@RNP^N^ and DOX@siRNP could be considered potential nanomedicines for cancer treatments.
